# Supplementary material for: Comparison of Circulating Cell-Free DNA Extraction Methods for Downstream Analysis in Cancer Patients
Source: Cancers (Basel). 2020 May 13;12(5):1222. doi: 10.3390/cancers12051222 (PMC7281769; doi:10.3390/cancers12051222)
Supplement: Supplementary file 1 [file cancers-12-01222-s001.pdf]

Supplementary Materials:

Comparison of Circulating Cell-Free DNA Extraction Methods for Downstream Analysis in Cancer Patients

Paul van der Leest, Pieter A. Boonstra, Arja ter Elst, Léon C. van Kempen, Marco Tibbesma, Jill Koopmans, Anneke Miedema, Menno Tamminga, Harry J. M. Groen, Anna K. L. Reyners and Ed Schuuring

Table S1. Sample details and malignancy status of included patients.

Sample ID	Source	Malignancy	Mutated Gene	Amino Acid Change
1	BCT-plasma	NSCLC	TP53	*p*.Y205C[2]
2	BCT-plasma	NSCLC	TP53	*p*.R273H[2]
3	EDTA-plasma	GIST	PDGFRA	*p*.M844_D846del[1]
4	EDTA-plasma	GIST	KIT	*p*.Y503_F504insAY[1]
5	EDTA-plasma	GIST	PDGFRA	*p*.I843_D846del[1]
6	BCT-plasma	NSCLC	No	-
7	BCT-plasma	NSCLC	No	-
8	BCT-plasma	NSCLC	Unknown	-
9	BCT-plasma	NSCLC	No	-
10	BCT-plasma	NSCLC	No	-
11*	BCT-plasma	NSCLC	BRAF	*p*.V600E[1]
12*	BCT-plasma	NSCLC	BRAF	*p*.V600E[1]
13*	BCT-plasma	NSCLC	BRAF	*p*.V600E[1]
14	BCT-plasma	NSCLC	KRAS	*p*.G13C[1]
15	BCT-plasma	NSCLC	KRAS	*p*.G12V[1]
16	BCT-plasma	NSCLC	BRAF	*p*.G466V[2]
17	EDTA-plasma	GIST	KIT	*p*.A502_Y503dup[1]
18	EDTA-plasma	GIST	KIT	*p*.A502_Y503dup[1]
19	EDTA-plasma	GIST	KIT	*p*.A502_Y503dup[1]
20ǂ	EDTA-plasma	GIST	No	-
21ǂ	EDTA-plasma	GIST	No	-
22	Citrate-plasma	NSCLC	KRAS	*p*.G12C[2]
23	Citrate-plasma	NSCLC	EGFR	*p*.N771_H773dup[2]
24	Citrate-plasma	NSCLC	No	-
25	Citrate-plasma	NSCLC	TP53	*p*.R273H[2]
26	Citrate-plasma	NSCLC	TP53	*p*.H179R[2]
27	Citrate-plasma	NSCLC	KRAS	*p*.G12D[2]

Highlighted are the cases for which a mutation-specific ddPCR analysis has been performed in the twenty-one cancer patients plasma cohort (blue). In the ccfDNA of cases 11, 12 13 14 and 15, no mutant droplets were detected with ddPCR. In the high-volume citrate plasma samples of cases 22, 25, 26 and 27, a mutation has been determined with a mutation-specific ddPCR analysis (green). BCT-plasma: plasma from cell free BCT tubes, EDTA-plasma: plasma from EDTA tubes, Citrate-plasma: citrate plasma retrieved through diagnostic leukapheresis (DLA), NSCLC: non-small cell lung cancer, GIST: gastro-intestinal stromal tumor, [1]mutation detected with tissue NGS, [2]mutation detected with plasma NGS, *samples derived from the same patient at different timepoints during anticancer treatment, ǂsamples derived from the same patient.

Table S2. Short- and medium-sized fragment percentages as measured with the Fragment Analyzer.

Kit	Fragment Analyzer	β-Actin One-Tube 3-Size ddPCR	
	**Ratio S/M**	**Ratio 137/420bp**	**Ratio 137/1950bp**
CNA	3.51 (2.88–4.43)	2.22 (1.84–3.02)	17.0 (10.9–22.6)
RSC	4.09 (3.24–5.20)	2.51 (1.92–3.63)	19.0 (13.1–51.7)
Z	3.54 (2.62–4.72)	3.00 (1.38–3.74)	6.51 (4.43–18.5) *

Measurements are displayed as median percentage of retrieved fragment size with the interquartile range within brackets. Ratio S/M: ratio between short-sized fragments (50–250bp) and medium-sized fragments (250-450bp). * $p < 0.05$ between RSC and Z.

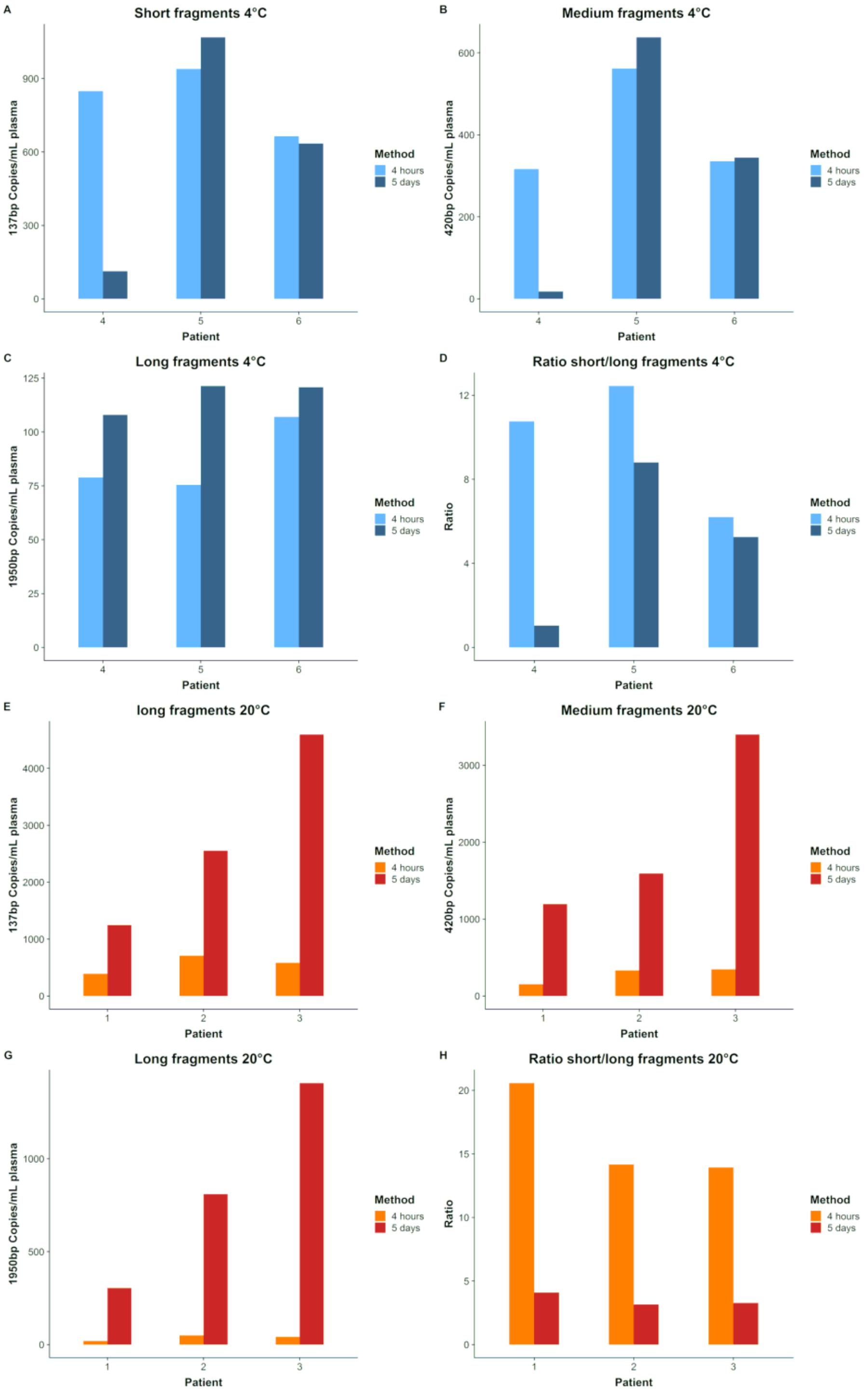

Figure S1. Detection of plasma ccfDNA degradation after short or long storage at 4 °C and 20 °C. In order to evaluate the degree of ccfDNA degradation in plasma from EDTA-tubes, six samples were processed in duplicate, one within 4 hours (standard procedure) and the second after 5 days of venipuncture. Half of these samples were stored at 4 °C and the other half at room temperature (20 °C; three samples per temperature). All these samples were isolated with the QIAamp Circulating Nucleic Acid kit and evaluated with the β-actin one-tube 3-size ddPCR assay.Bar graphs illustrating the degradation of plasma ccfDNA after 5 days of storage before extraction compared to extraction within 4 hours of venipuncture. Storage at 4 °C for the 137 bp **(A)**, 420 bp **(B)**, and 1950 bp **(C)** fragment sizes and the 137/1950 bp ratio **(D)**, as well for storage at 20 °C **(E-H)** are displayed. Two out of three plasma samples showed minimal ccfDNA degradation when stored at 4 °C. All samples stored at 20 °C showed an increase in total ccfDNA level. However, this is mainly in long-sized fragments, represented by the decreased short-to-long-ratio.

Table S3. Amplifiability of the extracted ccfDNA among different extraction kits.

Kit	137 bp (Copies/mL)	420 bp (Copies/mL)	1950 bp (Copies/mL)	137 bp (Copies/ng)	420 bp (Copies/ng)	1950 bp (Copies/ng)
CNA – blood plasma	637 (276-1658)	257 (140-587)	31 (22-129)	41 (27-53)	18 (13-22)	2.5 (1.6-3.2)
RSC – blood plasma	453 (254-832)	184 (125-269)	21 (9.2-29)	48 (30-65)	16 (12-23)	1.4 (0.4-2.8)
Z – blood plasma	203 (73-634)	106 (44-207)	25 (13-48)	21 (9.4-38)	10 (4.6-16)	2.4 (0.9-4.1)
CNA – blood plasma	288 (226-360)	146 (106-179)	55 (28-75)	35 (27-53)	20 (16-24)	5.7 (2.8-7.4)
ME – blood plasma	202 (181-227)	105 (95-126)	29 (23-38)	77 (61-81)	43 (38-50)	9.1 (7.0-12)

Highlighted are the cases results of the twenty-one cancer patients plasma cohort (blue) and of the six cancer patients high volume citrate plasma cohort (green). Measurements are displayed as median percentage of retrieved fragment size with the interquartile range within brackets. CNA – blood plasma: CNA-extracted blood plasma samples, RSC – blood plasma: RSC-extracted blood plasma samples, Z – blood plasma: Z-extracted blood plasma, CNA – citrate plasma: CNA-extracted blood citrate samples samples, ME – blood plasma: ME-extracted citrate plasma samples, copies/mL: copies per initial mL of plasma input, copies/ng: copies per ng of ccfDNA.

Table 4. Sequences of the applied primers and probes.

Assay	Forward Primer	Reverse Primer	Probe	Assay ID
137bp	5′-GCGCCGTTCCGAAAGTT-3′	5′-CGGCGGATCGGCAAA-3′	5′-ACCGCCGAGACCGCGTC-3′	-
420bp	5′-CCGCTACCTCTTCTGGTG-3′	5′-GATACACCATGTCACACT-3′	5′-CCTCCCTCCTTCCTGGCCTC-3′	-
KRAS G12/G13 screening	-	-	-	1863506
BRAF *p*.G466V	-	-	-	dHsaMDS2510966
TP53 *p*.H179R	-	-	-	dHsaMDV2510520
PDGFRA *p*.M844_D846del	5′-CTCCTGGCACAAGGAAA-3′	5′-AAAGGCAGTGTACGTCC-3′	5′-TGGCCAGAGACATCAACTATGTGTCG-3′	-
TP53 *p*.R273H	-	-	-	dHsaMDV2010109
TP53 *p*.Y205C	-	-	-	dHsaMDV2516922
